# pSiM24 Is a Novel Versatile Gene Expression Vector for Transient Assays As Well As Stable Expression of Foreign Genes in Plants

**DOI:** 10.1371/journal.pone.0098988

**Published:** 2014-06-04

**Authors:** Dipak Kumar Sahoo, Nrisingha Dey, Indu Bhushan Maiti

**Affiliations:** 1 KTRDC, College of Agriculture, Food and Environment, University of Kentucky, Lexington, Kentucky, United States of America; 2 Department of Gene Function and Regulation, Institute of Life Sciences, Bhubaneswar, Odisha, India; Institute of Genetics and Developmental Biology, Chinese Academy of Sciences, China

## Abstract

We have constructed a small and highly efficient binary Ti vector pSiM24 for plant transformation with maximum efficacy. In the pSiM24 vector, the size of the backbone of the early binary vector pKYLXM24 (GenBank Accession No. HM036220; a derivative of pKYLX71) was reduced from 12.8 kb to 7.1 kb. The binary vector pSiM24 is composed of the following genetic elements: left and right T-DNA borders, a modified full-length transcript promoter (M24) of *Mirabilis mosaic virus* with duplicated enhancer domains, three multiple cloning sites, a 3′rbcsE9 terminator, replication functions for *Escherichia coli* (ColE1) and *Agrobacterium tumefaciens* (pRK2-OriV) and the replicase trfA gene, selectable marker genes for kanamycin resistance (nptII) and ampicillin resistance (bla). The pSiM24 plasmid offers a wide selection of cloning sites, high copy numbers in *E. coli* and a high cloning capacity for easily manipulating different genetic elements. It has been fully tested in transferring transgenes such as green fluorescent protein (GFP) and β-glucuronidase (GUS) both transiently (agro-infiltration, protoplast electroporation and biolistic) and stably in plant systems (*Arabidopsis* and tobacco) using both agrobacterium-mediated transformation and biolistic procedures. Not only reporter genes, several other introduced genes were also effectively expressed using pSiM24 expression vector. Hence, the pSiM24 vector would be useful for various plant biotechnological applications. In addition, the pSiM24 plasmid can act as a platform for other applications, such as gene expression studies and different promoter expressional analyses.

## Introduction

The transfer of foreign genes into higher plants mediated either by *Agrobacterium tumefaciens* or by employing a biolistic process is the core technique used in genetic engineering-based plant modification. Many useful and versatile vectors have been constructed since the birth of the concept and the first generation of binary vectors for plant transformation [1]–[6]. The general trend in the binary vector development has been to increase the plasmid stability during a long co-cultivation period of *A. tumefaciens* with the target host plant tissues and also to understand the molecular mechanism of broad host-range replication, and to use it to reduce the size of plasmid for ease in cloning and for higher plasmid yield in *Escherichia coli* [7], [8]. A number of large (>10 kb), first-generation binary vectors have been constructed for plant transformation, including Ti plasmid [2], pBin19 [1], pKYLX7 [4] and other expression vectors [3]. One of the binary vectors, pBin19 [1], has been modified to pBI121 and pIG121Hm [9], [10] to use the β-glucuronidase (*GUS*) reporter gene in plant transformation. Binary vectors include pKYLX expression vectors containing 35S and rbcS promoters that are suitable for constitutive or light-regulated expression of foreign genes [4]. These vectors and their derivatives were soon widely distributed among plant scientists. In addition, another widely used series of vectors includes pPZP vectors [11] and their modified form, pCAMBIA vectors (www.cambia.org). Xiang et al., (1999) constructed a pCB mini-binary vector series [12] from the relatively large, first-generation binary plasmid pBin [1]. Over time, vector technology evolved, and new generations of plant transformation vectors with improved cloning and delivery strategy were introduced, for example, pGreen vectors [13]; pGD or pSITE vectors, which are suitable for the stable integration or transient expression of various autofluorescent protein fusions in plant cells [14], [15]; the pCLEAN binary vector system [16]; the pHUGE binary vector system [17]; and binary bacterial artificial chromosome BIBAC vectors [18]. The TMV RNA-based vector pJLTRBO [19]and its derivative pPZPTRBO [20] were reported to produce recombinant proteins in plants without using the RNA-silencing inhibitor P19. Similar expression levels were provided by the pEAQ-HT vector which has an integrated P19 expression cassette [21]. A bean yellow dwarf virus single-stranded DNA-based vector, pBY030-2R was reported to produce high amount of recombinant proteins [22] while the pMAA-Red vector was known for easy production of transgenic *Arabidopsis* overexpression lines with strong expression levels of the gene of interest [23].

The binary vectors widely used for plant transformations vary in size, origin of replication, bacterial selectable markers, T-DNA borders and overall structure. Recent modifications of binary vectors provide a number of user-friendly features, such as a wide selection of cloning sites, high copy numbers in *E. coli*, improved compatibility with strains of choice, a wide pool of selectable markers for plants and a high frequency of plant transformation. Although recent improvements are very useful, the classic vector configuration still appears to be good enough in many occasions. Plasmid manipulations are also easier if the vector replicates in *E. coli* to high copy numbers. Moreover, the efficiency of *in vitro* recombination procedures is inversely proportional to the size of the vector DNA [24]. With an increased requirement for the transfer of large pieces of DNA into plants, the size of binary vectors should be kept to a minimum. The availability of low-molecular-weight, versatile plant expression vectors is currently insufficient in plant molecular biology. For these reasons, we designed a smaller binary vector, pSiM24, which offers a wide selection of cloning sites, high copy numbers in *E. coli* and is fully functional in the transient (using both the gene-gun or Agro-infiltration methods) as well as stable transformation of plants.

## Materials and Methods

### Chemicals, enzymes, bacterial strains and plasmids

Antibiotics (ampicillin, kanamycin, rifampicin, tetracycline, hygromycin) and chemicals were purchased from Sigma-Aldrich (St. Louis, MO, USA) or Thermo Fisher Scientific (Waltham, MA, USA). All restriction endo-nucleases and DNA-modifying enzymes were obtained from New England Biolab (Beverly, MA, USA) or Invitrogen-Life Technologies (Grand Island, NY USA). The TB1 strain of *E. coli* [25] and the C58C1 [GV3850] strain of *A. tumefaciens* [26] were used. The plasmids pBluescriptIIKS(+) (Genbank Accession no. X52327) from Stratagene (la Jolla, CA, USA) and pKYLX7 [4] or its derivative pKM24KH (GenBank accession no. HM036220.1) were used. Cultures of *E. coli* transformed with pUC-based vectors were grown in the presence of ampicillin (100 µg/ml). Transformed agrobacterium was grown in the presence of kanamycin (25 µg/ml) and rifampicin (100 µg/ml). The Vip3A and KMP-11 antibodies were provided by Dr Raj K. Bhatnagar, ICGEB, New Delhi, India and Dr Shyamal Roy, IICB, Kolkata, India. The IL-10 antibody was obtained from Imgenex, Bhubaneswar, India.

### In vitro cloning procedures and DNA sequencing

All in vitro recombination techniques were employed using previously described standard methods [27], [28]. For DNA sequencing, a dye terminator labeling procedure was followed using a Genome Lab DTCS-Quick Start kit (Beckman Coulter, USA), and an automated sequencing machine (Beckman Coulter CEQ 8000 Genetic Analysis System, USA) was used in accordance with the manufacturer's instructions.

### Construction of plasmid vector pBTdna, pBTdna-rbcT and pBTdna-rbcT-KanR

We designed and generated a 522-bp synthetic DNA fragment containing left and right T-DNA borders and three multiple cloning sites (MCS) of general the structure 5′-BssHI-KpnI-left T-DNA(147-bp)-MCS1(BstXI-StuI-FspAI-PasI-SanDI-BstZ171-SmaI)-MCS2 (EcoRI-HindIII-BamHI-XhoI-HpaI-MluI-SalI-SstI-PstI-XbaI)-MCS3 (ClaI-SpeI-BglII-BstEII-EcoNI-FseI-SwaI-NruI-PacI-right T-DNA border (162-bp)-EcoRV-BssHII-3′. This fragment was synthesized by GenArt-Life Technologies (Carlsbad, CA, USA). The 5′-BssHII-BssHII-3′fragment was cloned into the corresponding sites of pBluescriptIIKS(+), and the resulting plasmid was named pBTdna.

A 657-bp poly(A) signal 3′-rbcS-E9 of the general structure 5′-ClaI-3′-rbcSE9-XbaI-3′ was isolated from the binary vector pKM24KH (GenBank Accession No. HM036220) and was inserted into the corresponding site of pBTdna to generate the plasmid pBTdna-rbcT.

A 1343-bp synthetic neomycin phosphotransferase gene/kanamycin resistance gene (nptII/KanR) of the general structure 5′-BglII-Nos-promoter-KanR cDNA-Nos-terminator-SpeI-3′ was obtained from GenArt-Life Technologies (Carlsbad, CA, USA). The open reading frame of the KanR gene was optimized for plant codon bias. The KanR gene, with the structure 5′-BglII-SpeI-3′, was cloned into the corresponding sites of pBTdna-rbcT to create the plasmid pBTdna-rbcT-KanR (also called pBTRK). The plasmid contains a 2498-bp micro-Tdna fragment of the general structure 5′-BssHII-KpnI-left T-DNA border-MCS1-MCS-2-XbaI-3′rbcS Terminator-ClaI-SpeI-KanR gene (complement)-BglII-MCS3-right T-DNA border- EcoRV-BssHII-3′. The sequence integrity of the fragment was confirmed before further use. The sequence information of these genetic elements is provided in the NCBI database (GenBank accession no. KF032933).

### Construction of non-T-DNA plasmids: pBtrfA, pB-oriV-trfA, pBAmpR-ColEI-oriV-trfA

We designed a non-T-DNA plasmid of the general structure 5′-BssHII-KpnI-AmpR gene-ColE1 (origin of replication of pMB1)-ApaI-oriV of pRK2-SalI-trfA gene-EcoRV-BssHII-3′. A synthetic DNA fragment of the physical map 5′-BssHII-KpnI-ApaI-SalI-trfA gene-EcoRV-BssHII was obtained from a commercial supplier (GenArt-Life Technologies, CA, USA). The open reading frame of the trfA gene was optimized with the bias codon of *A. tumefaciens*. This 5′-BssHII-BssHII-3′ fragment was cloned into the corresponding sites of pBluescriptIIKS(+) to create the plasmid pBtrfA.

A 642-bp fragment of the replicon OriV of pRK2 was PCR-amplified using appropriately designed forward and reverse primers to insert an ApaI site at the 5′-end and a SalI site at the 3′-end. The gel-purified PCR fragment 5′-ApaI-OriV-SalI-3′ was inserted into the corresponding site of pBtrfA to form the plasmid pB-oriV-trfA. A fragment of 1803 bp containing the AmpR gene and the ColE1 replicon in pMA (GeneArt vector, Registry part no. K157000), a pUC derivative, was PCR-amplified using appropriately designed forward and reverse primers to insert the KpnI site at the 5′-end and the ApaI site at the 3′-end. The PCR fragment was digested with KpnI and ApaI, and the gel-purified fragment 5′-KpnI-ApaI-3′ was cloned into the corresponding site of pB-oriV-trfA to generate the plasmid pBAmpR-ColEI-oriV-trfA.

### Construction of pSi and pSiM24

The T-DNA portion was isolated from pBTdna-rbcT-KanR. First, the pBTdna-rbcT-KanR plasmid was digested with PvuII; the larger band (4235-bp fragment) was isolated and further digested with BssHII to generate a 2492-bp fragment of the general structure (5′-BssHII-KpnI-left T-DNA border-MCS-1-MCS-2-XbaI-3′rbcS Terminator-ClaI-MCS-3-right T-DNA border-EcoRV-BssHII-3′). The non-T-DNA portion of a 3830-bp fragment of the general structure (5′-BssHII-KpnI-AmpRI-ColEI-ApaI-OriV-SalI-trfA-EcoRV-BssHII-3′) was isolated from pBAmpR-ColEI-oriV-trfA. Two fragments (T-DNA and nonT-DNA portions) were ligated and circularized to produce the binary vector pSi. The modified full-length transcript promoter (M24) of the *Mirabilis mosaic virus* [27], [29] was inserted as 5′-EcoRI-HindIII-3′ into the corresponding sites of pSi, and the resulting plasmid was named pSiM24. The fully annotated sequence of pSiM24 is available in the NCBI database (GenBank accession no. KF032933).

### Construction of plant expression vectors with green fluorescent protein (GFP) and β-glucuronidase (GUS) reporter genes

The M24 promoter fragment 5′-EcoR1-M24-HindIII-3′ and the reporter gene 5′-XhoI-GUS-SstI-3′ or 5′-XhoI-GFP-SstI-3′ were inserted into the corresponding sites of pBTRK and pSi to generate expression constructs pBTRK-M24-GUS/GFP and pSiM24-GUS/GFP, respectively.

### Tobacco plant transformation

The plant expression constructs pSiM24-GUS and pSiM24 were introduced into the *A. tumefaciens* strain GV3850 by the freeze-thaw method [30]. Tobacco plants (*Nicotiana tabacum* cv. SamsunNN) were transformed with *Agrobacterium* harboring pSiM24-GUS and pSiM24 constructs as described previously [31] or by the gene-gun method using pSiM24-GUS and pSiM24 constructs [32]. Tobacco shoots and then roots were regenerated from kanamycin-resistant calli derived from independent leaf discs. Ten independent kanamycin-resistant plant lines (R_0_ generation, 1^st^ progeny) were generated for the constructs pSiM24-GUS and pSiM24 and were maintained under greenhouse conditions (30±5°C with both natural and supplementary lighting of minimum photon flux density, 300 µmole/m^2^/s, 17 h day/7 h night cycle). Seeds were collected from self-pollinated primary transformants. Transgenic tobacco seeds (R1 progeny, 2^nd^ generation) were germinated in the presence of Kanamycin (250 mg/L). Positive transformants with a KanR:KanS ratio of 3∶1 progeny segregation were selected for further analysis. Transgenic lines (R_1_ and R_2_ progeny, second and third generation) were screened for gene integration, transcription and translation by polymerase chain reaction (PCR), reverse transcriptase-PCR (RT-PCR), real-time quantitative RT-PCR (qRT-PCR), enzymatic assays and GUS histochemical analysis.

### Generation of transgenic *Arabidopsis* plants

The pSiM24 and pSiM24-GUS plasmids introduced into *A. tumefaciens* GV3850 were used to transfer each of these constructs into *Arabidopsis* (*Arabidopsis thaliana* ecotype Columbia-0) by the floral dip method [33]. The transgenic *Arabidopsis* plants were selected and maintained as described previously [34].

### Transient Agro-infiltration assay of pSiM24-GUS in tobacco leaves

Suspensions of the *A. tumefaciens* strain GV3850 bearing pSiM24 and pSiM24-GUS constructs were infiltrated into leaves of *Nicotiana benthamiana* as described previously [35]. After two days of agro-infiltration, the transient GUS expression was evaluated by the histochemical GUS staining method [9].

### Transient expression analysis in tobacco protoplasts

The isolation of tobacco protoplasts from the suspension cell cultures of *N. tabacum* L. cv Xanthi-Brad and electroporation of tobacco protoplasts with supercoiled plasmid pBTRKM24-GUS/GFP and pBTRKM24 constructs were performed as described previously [36]. After 20 h, protoplasts were harvested for fluorometric GUS enzymatic assay [9]. GUS expression levels were within ±10% for a given construct in this study. All constructs were tested in at least five independent experiments.

### Biolistic-onion peel transient assay

Onion tissues were prepared and bombarded with pBTRKM24, pBTRKM24-GUS, pSiM24 and pSiM24-GUS plasmids following a standard protocol [37]. After two days, transient GUS expression was detected by a histochemical method [9] and visualized under an Olympus SZX12 bright-field microscope.

### Real-time quantitative reverse transcription polymerase chain reaction (qRT-PCR)

The expression levels of GUS mRNA in transgenic tobacco and *Arabidopsis* plants developed for the plasmids pKCaMV35SGUS and pSiM24GUS were evaluated by real-time quantitative RT-PCR [38] using GUS-specific forward (5′-d-TTACGTCCTGTAGAAACCCCA-3′) and reverse (5′-d-ACTGCCTGGCACAGCAAT TGC-3′) primers. The qPCR assays were performed using the iTaq SYBR Green Supermix with ROX (Bio-Rad, USA) according to the manufacturer's instructions. Tobacco tubulin (by using forward 5′-d-ATGAGAGAGTGCATATCGAT-3′ and reverse 5′-d-TTCACTGAAGAAGGTGTTGAA-3′ primers) was used as an internal control to normalize the expression of GUS. The comparative threshold cycle (Ct) method (Applied Biosystems bulletin, part No. 4376784 Rev. C, 04/2007) was used to evaluate the relative expression levels of the transcripts. The threshold cycle was automatically determined for each reaction by the system set with default parameters (Step One Real-Time PCR System, Applied Biosystems). The specificity of the PCR was determined by melting curve analysis of the amplified products using the standard method installed in the system (Step One Real-Time PCR System, Applied Biosystems).

### β-Glucuronidase (GUS) assay and histochemical GUS staining

Fluorometric GUS enzymatic assays for measuring GUS activities in tobacco protoplast extracts, *Arabidopsis* and tobacco plant extracts were performed as described previously [9], [39]. The total protein content in protoplast and plant extracts was estimated by the Bradford method using BSA as a standard [40]. Histochemical GUS staining was carried out in plants following the published protocol [9], [34], and photographs were taken under a bright-field microscope (Olympus SZX12).

### GFP detection

GFP fluorescence images of electroporated tobacco protoplasts, onion epidermal cells and transgenic *Arabidopsis* leaves expressing GFP were analyzed using a confocal laser scanning microscope (TCS SP5; Leica Microsystems CMS GmbH, D-68165 Mannheim, Germany) using LAS AF (Leica Application Suite Advanced Fluorescence) 1.8.1 build 1390 software under a PL FLUOTAR objective (10.0X/N.A.0.3 DRY) using a confocal pinhole set of 1 airy unit and a 1× zoom factor for improved 8-bit resolution, as described previously [28], [38]. To excite the expressed GFP in transgenic plants, a 488-nm argon laser (30%) with an AOTF (allowing for 40% transmission) was used, and fluorescence emission spectra were collected between 501 and 580 nm with the photomultiplier tube (PMT) detector gain set to 1050 V [28].

### Transient expression of GUS using pSiM24 vector through vacuum infiltration method

Suspensions of the *A. tumefaciens* strain GV3850 bearing pSiM24 and pSiM24-GUS constructs were prepared as previously described [35], and the infiltration procedure was conducted following a previously reported protocol [41]. Leaves of *N. benthamiana* were weighed and submerged in a suspension of *A. tumefaciens* strain GV3850 bearing pSiM24 or pSiM24-GUS plasmids. A vacuum level of 760 mm Hg was applied and released several times until the leaves became translucent [41]. Leaves were transferred into MS-media-containing plates and incubated at room temperature for two days. GUS expression in the infiltrated leaves was evaluated by the GUS histochemical staining method and GUS assay [9], [39].

### Analysis GFP*-AtCESA3^ixr1-2^*, Vip3A(a), KMP-11, IL-10 and nat-T-phyllo-GFP after transient expression in tobacco using pSiM24 vector

The GFP fused *Arabidopsis* mutated CESA3 (GFP*-AtCESA3^ixr1-2^*) fragment with Xho I and Sst I sites was obtained from pKM24KH-MD1 (GenBank accession no. JX996118) [42]–[43] by restriction digestions. Likewise, the native T-phylloplanin fused GFP with the apoplast targeting sequence (nat-T-phyllo-GFP) fragment with Xho I and Sst I sites was obtained from pKM24-ibm8 (GenBank accession no. KF951257) [44] by restriction digestions. Both these fragments were cloned in pSiM24 following standard protocols [28] and the resulted plasmids were named as pSiM24-GFP*-AtCESA3^ixr1-2^* and pSiM24-nat-T-phyllo-GFP. Suspension of *A. tumefaciens* strain pGV3850 harboring pSiM24, pSiM24-GFP*-AtCESA3^ixr1-2^* and pSiM24-nat-T-phyllo-GFP constructs were infiltrated into leaves of tobacco plants (*N. tabacum* cv. SamsunNN) as described earlier [35]. After two days of Agro-infiltration the transient *AtCESA3^ixr1-2^* expression was evaluated by RT-PCR by using gene specific primers and also by Western blotting using *AtCESA3^ixr1-2^* polyclonal antibody as described earlier [42]. The transient nat-T-phyllo-GFP expression was evaluated by RT-PCR by using gene specific primers and also by confocal microscopy as previously described [44].

Vegetative insecticidal gene, *vip3A(a)* [45], [46], kinetoplastid membrane protein-11 (KMP-11) [47] and interleukin-10 (IL-10) [48] were cloned at XhoI and SstI sites in pSiM24 vector to generate pSiM24-vip3A(a), pSiM24-KMP-11 and pSiM24-IL-10 plasmids for transient expression assay in tobacco protoplasts. The isolation of tobacco protoplasts from the suspension cell cultures of *N. tabacum* L. cv Xanthi-Brad and electroporation of tobacco protoplasts with pSiM24-vip3A(a), pSiM24-KMP-11 and pSiM24-IL-10 constructs were performed as described previously [28]. Electroporated protoplasts were incubated for 48 hours and harvested in protein extraction buffer (1X PBS, 0.1% Tween 20 and 1 mM PMSF). Protein samples from pSiM24, pSiM24-vip3A(a), pSiM24-KMP-11 and pSiM24-IL-10 transfected protoplasts were lyophilized and dissolved in protein extraction buffer. The transient *Vip3A(a)* expression was evaluated by Western blotting using Vip3A-specific polyclonal antibody as described earlier [46]. Concentration of transiently expressed KMP-11 and IL-10 was estimated by using anti-KMP-11 and anti-IL-10 antibody following indirect enzyme-linked immunosorbance assay (ELISA) protocol [49].

## Results

### Features of assembled binary expression vector pSiM24

**Figure 1:**
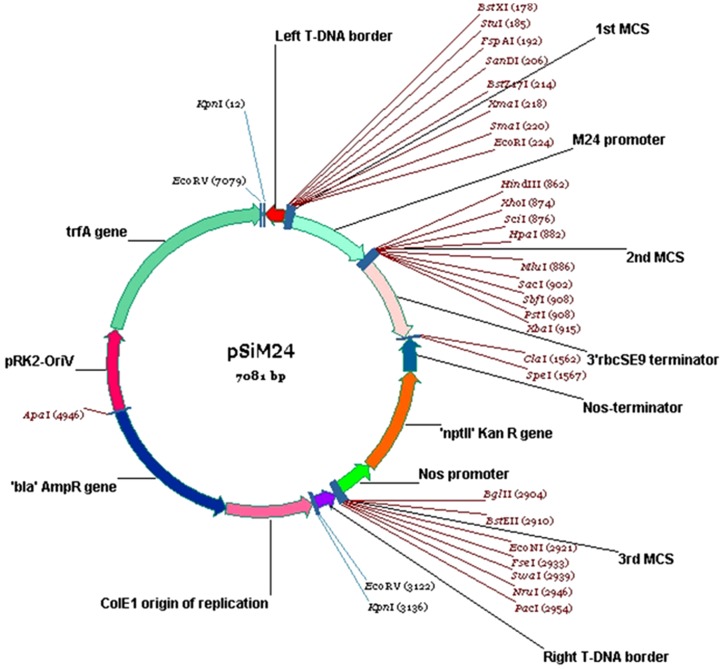
Schematic presentation of binary vector pSiM24. The backbone structure of binary vector pSiM24 (7081-bp) containing the modified full-length transcript promoter (M24) of the *Mirabilis mosaic virus*, which directs the coding sequences of the gene of interest; left T-DNA and right T-DNA borders (Left T-DNA, Right T-DNA); selection marker genes (KanR, neomycin phosphotransferase II, nptII) directed by nopaline synthase promoter (Nos promoter); terminator sequences of ribulose bisphosphate carboxylase small subunits (3′rbcSE9); nopaline synthase terminator (Nos terminator); multiple cloning sites (first MCS, second MCS and third MCS) with various restriction sites; replicon unit pRK2 oriV; trfA gene for agrobacterium; ColE1 origin of replication for *E. coli*; and ‘bla’ AmpR gene for resistance to ampicillin.

The binary expression vector pSiM24 was designed to reduce the size of the vector backbone by eliminating non-essential elements of our previous vector pKM24KH (size 12,945-bp, GenBank accession no. HM036220), a derivative of pKYLX7 [4]. The pKM24KH vector is a low-copy-number plasmid. We replaced the *E. coli* replication unit with a high-copy-number replicon ColEI in pSiM24, making the identification and characterization of gene inserts easier. We also modified the agrobacterium replicon unit (Oriv-trfA of pRK2) by optimizing the trfA open reading frame for better expression. The overall DNA yields and transformation frequency of the new vector pSiM24 were several times greater than those of the previous vector pKM24KH in both *E. coli* and *A. tumefaciens*. The binary vector pSiM24 (Figure 1; GenBank Accession no. KF032933) has the following genetic elements: left T-DNA border (coordinates 65 to 90, complement); M24 promoter (223 to 860); three multiple cloning sites; 3′rbcS terminator (915 to 1565); KanR(nptII) gene (1566 to 2911, complement); KanR-terminator (1566 to 1820, complement); Kan R-cDNA (1821 to 2618, complement); KanR-promoter (2619 to 2903, complement); right T-DNA (2957 to 3118), right T-DNA border (3034 to 3059, complement); non-T-DNA portion, ColE1 origin of replication (3137 to 3804, complement); AmpR(bla) gene region (3805 to 4940, complement); terminator (3805 to 3951, complement); AmpR-cDNA (3952 to 4812, complement); AmpR-promoter (4813 to 4940, complement); pRK2-Ori V (coordinates 4947 to 5567); pRK2-trfA gene (5577 to 7081); promoter (5577 to 5880); cDNA (5881 to 7029) and terminator (7030 to 7081).

### DNA yield and transformation frequencies of *E. coli* and *A. tumefaciens* with pSiM24 binary vectors

DNA yield and transformation frequencies in *E. coli* and *A. tumefaciens* were evaluated and presented (Tables 1–3). Transformations were performed with equal molar amounts of each of the plasmids to normalize increasing plasmid size as previously described [50]. The transformation frequencies of pSiM24 vectors are four- to six-fold higher than pCAMBIA and pKM24KH vectors, in *E. coli* (Table 1). The transformation frequency of the pSiM24 vector is 1.4- to 1.8-fold higher than conventional pCAMBIA and pKM24KH vectors, in *A. tumefaciens*, although the effect is not as marked as in *E. coli* (Table 2). The DNA yields of pSiM24 vectors were approximately three-fold greater than those of the pCAMBIA vector and seven to eight-fold higher than those of pKM24KH in *E. coli* (Table 3).

**Table 1:** Transformation frequencies of *Escherichia coli* strain TB1 for pSiM24 binary vector.

Plasmid	Total size (kb)	Amount DNA used (pg)	Average colony (n>4)	Ratio to pCAMBIA control	Ratio to pKM24KH control
pSiM24	7.08	100	634±54^b^	4.73	5.66
pSiM24-GUS	8.89	125	619±46^b^	4.62	5.53
pSiM24-GFP	7.8	110	608±38^b^	4.54	5.43
pCAMBIA2300	8.74	123.4	134±12^a^	1	1.2
pKM24KH	12.94	183	112±13^a^	0.84	1

The bacteria were transformed with equal molar amounts of each of the plasmid DNA. Statistical analysis of the data was performed adopting one way ANOVA analysis (using GraphPad Prism version 5.01) and presented as the means ± S.D. A *P* value of less than 0.05 was considered significant indicated by different superscript letters.

**Table 2:** Transformation frequencies of *Agrobacterium tumefaciens* strain GV3850 for pSiM24 binary vector.

Plasmid	Total size (kb)	Amount DNA used (µg)	Average colony (n>4)	Ratio to pCAMBIA control	Ratio to pKM24KH control
pSiM24	7.08	1	546±42^b^	1.4	1.81
pSiM24-GUS	8.89	1.25	534±48^b^	1.37	1.77
pSiM24-GFP	7.8	1.1	542±36^b^	1.39	1.79
pCAMBIA2300	8.74	1.23	391±33^a^	1	1.29
pKM24KH	12.94	1.83	302±20^c^	0.77	1

The bacteria were transformed with equal molar amounts of each of the plasmid DNA. Statistical analysis of the data was performed adopting one way ANOVA analysis (using GraphPad Prism version 5.01) and presented as the means ± S.D. A *P* value of less than 0.05 was considered significant indicated by different superscript letters.

**Table 3:** Binary Ti vectors pSiM24 produced higher plasmid DNA yields in *Escherichia coli* strain TB1 over pCAMBIA.

Plasmid	Average DNA yield (µg) (n>3)	Ratio to pCAMBIA control	Ratio to pKM24KH control
pSiM24	113.5±21.3^b^	3.47	8.11
pSiM24-GUS	96.5±13.2^b^	2.95	6.89
pSiM24-GFP	105.7±16.3^b^	3.21	7.55
pCAMBIA2300	32.74±4.0^a^	1	2.34
pKM24KH	14±1.6^c^	0.43	1

Three single colonies of each plasmid constructs were grown for 16 hrs in 25 ml LB media with 100 mg/L ampicillin (for pSiM24) or 50 mg/L kanamycin (for pCAMBIA) or 15 mg/L tetracycline (for pKM24KH). Plasmid DNA was purified using QIA Midiprep columns. DNA yields represent the average of three independent samples. Statistical analysis of the data was performed adopting one way ANOVA analysis (using GraphPad Prism version 5.01) and presented as the means ± S.D. A *P* value of less than 0.05 was considered significant indicated by different superscript letters.

### Transient expression of the pBTRKM24-GUS and pSiM24-GUS/GFP constructs

**Figure 2:**
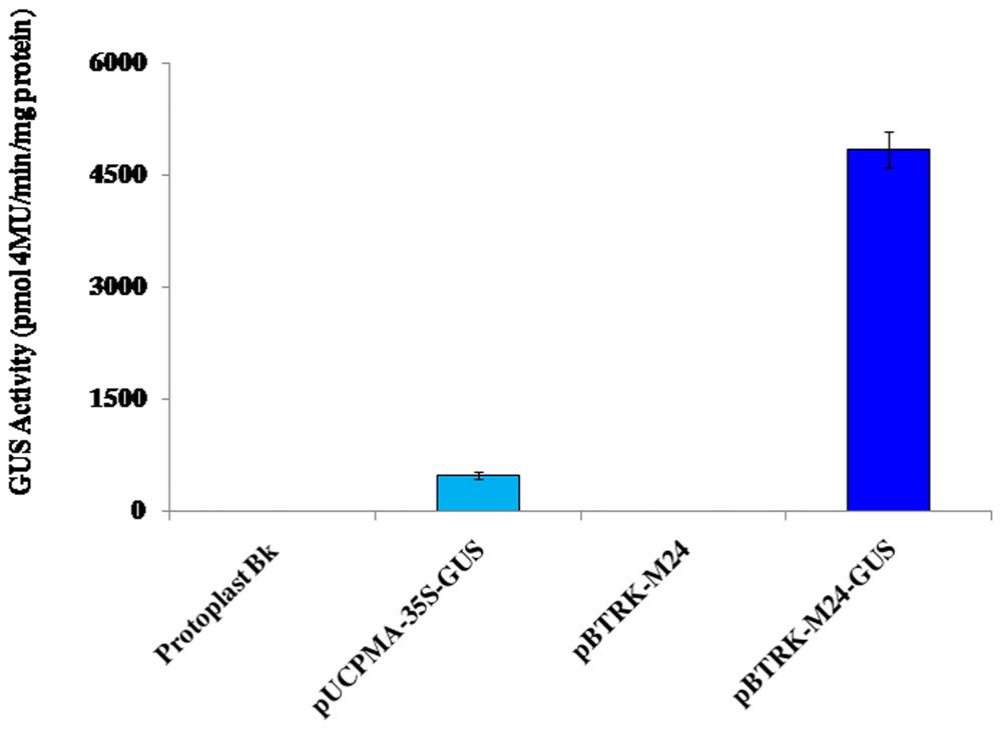
The transient GUS expression analysis of T-DNA assembled fragment of pSiM24 in tobacco protoplast system. Transient GUS expression analysis of pBTRK-M24 (T-DNA assembled fragment of pSiM24), pBTRK-M24-GUS (pBTRK-M24 with GUS reporter gene) constructs in tobacco protoplast. The pUCPMA-35S-GUS construct carries the constitutive CaMV35S promoter. The average GUS activity ± SD is presented in the histogram of three independent experiments replicated three times for each construct. The values significantly differ between tobacco protoplasts with pBTRK-M24-GUS from others at P<0.01 based on Student's *t*-test.

**Figure 3:**
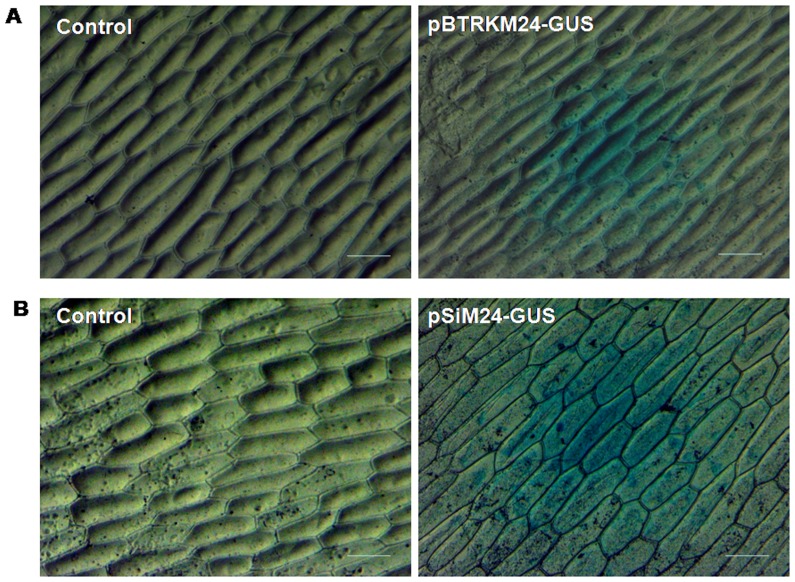
The transient GUS expression analysis of T-DNA assembled fragment of pSiM24 containing GUS and pSiM24-GUS in onion epidermal cells. (A) Light microscopy images of X-gluc-treated onion epidermal cells bombarded with pBTRKM24-GUS (T-DNA assembled fragment of pSiM24 with GUS reporter gene) construct DNA-loaded gold particles are presented. Control represents untransformed onion epidermal cells. These micrographs are representative of data collected after examination of onion epidermal cells from a minimum of four independent experiments. Scale bar, 100 µm. (B) Light microscopy images of X-gluc-treated onion epidermal cells bombarded with pSiM24-GUS (pSiM24 with GUS reporter gene) construct DNA-loaded gold particles are presented. Control represents onion epidermal cells with pSiM24 without GUS reporter gene. These micrographs are representative of data collected after examination of onion epidermal cells from a minimum of four independent experiments. Scale bar represents 100 µm.

The pBluescript-based constructs pBTRKM24-GUS with an M24 promoter and pUCPMA35S-GUS [27] with a 35S promoter were compared by tobacco protoplast transient assay. The M24 promoter showed approximately 10 times higher GUS activity than the CaMV 35S promoter (Figure 2). The pBTRKM24-GUS construct was also evaluated by the biolistic bombardment of epidermal cells of onion peels, showing strong GUS expression, as detected histochemically (Figure 3).

**Figure 4:**
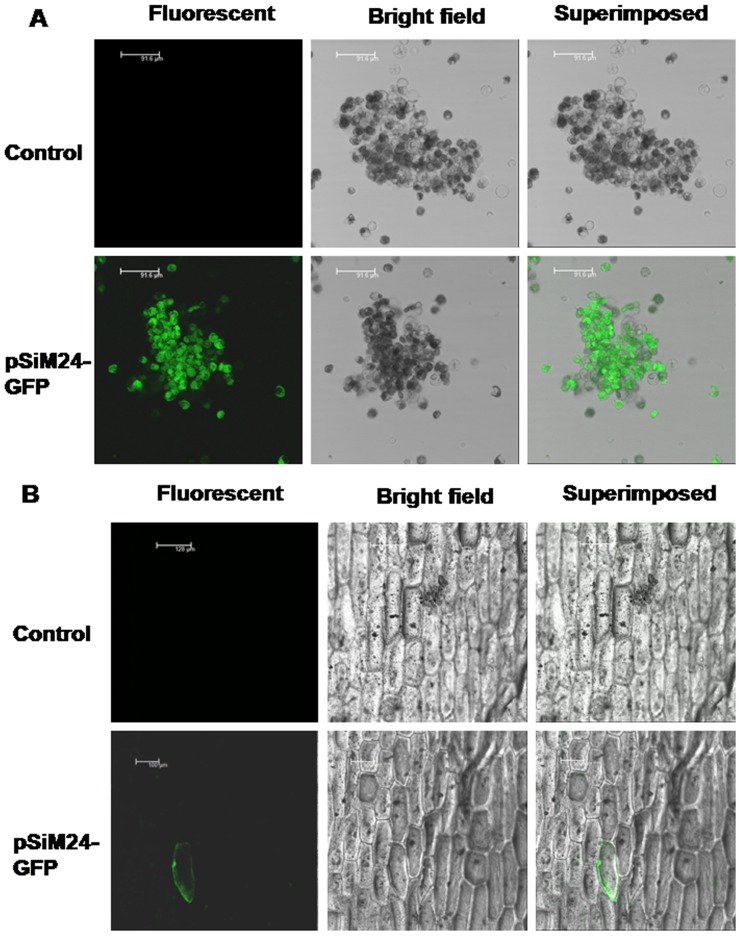
Transient expression of pSiM24-GFP in tobacco protoplasts and onion epidermal cells. (A) Protoplasts were transfected with plasmids pSiM24 (Control) and pSiM24-GFP (having GFP reporter gene). Transformation efficiencies were determined by analyzing the protoplasts with fluorescence after incubation for overnight. Fluorescent, bright-field and superimposed (bright-field and green fluorescent) confocal laser scanning micrographs of tobacco protoplasts are presented. Scale bar, 92 µm. (B) Fluorescent, bright-field and superimposed (bright-field and green fluorescent) confocal laser scanning micrographs of onion epidermal cells bombarded with pSiM24-GFP construct DNA-loaded gold particles are presented. Control represents onion epidermal cells with pSiM24 visualized under CLSM. Scale bar, 100 µm.

**Figure 5:**
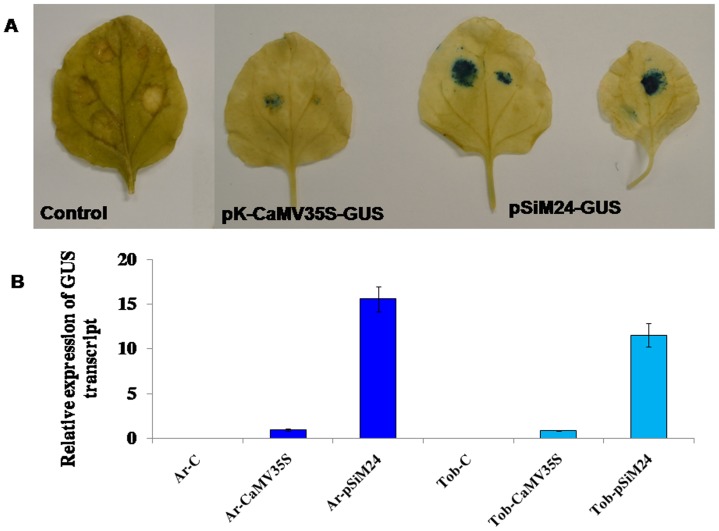
GUS expression analysis of pSiM24-GUS and pKCaMV35S-GUS in transient and transgenic systems. (A) Representative transient GUS expression levels from agrobacterium infiltration assays in *N. benthamiana* leaves are shown for pKCaMV35S-GUS and pSiM24-GUS constructs. GUS was detected histochemically. Control represents pSiM24 without GUS reporter gene. (B) Relative expression of GUS specific transcripts was measured in whole transgenic *Arabidopsis* (second generation, two weeks old; Ar-CaMV35S with pKCaMV35S and Ar-pSiM24 with pSiM24) and tobacco (second generation, three weeks old; Tob-CaMV35S with pKCaMV35S and Tob-pSiM24 with pSiM24) plants. The data represent relative expression of GUS transcript ± S.D. of four independent lines (n = 4) for each construct in which five plants per line were analyzed. The values significantly differ between control and transgenic plants at P<0.01 based on Student's *t*-test.

The pSiM24-GFP (with a different reporter gene, i.e., GFP) was studied in a tobacco protoplast system, where GFP fluorescence was visualized by confocal microscopy (Figure 4). The pSiM24-GUS construct was tested in an *Agrobacterium* infiltration assay in *N. benthamiana* leaves. The *A. tumefaciens* (strain C58C1-GV3850) carrying pSiM24 (empty vector), pK-CaMV35S-GUS and pSiM24-GUS constructs was used for agro-infiltration. Transient GUS expression detected histochemically, showed stronger GUS expression in agro-infiltrated patches for pSiM24-GUS construct than for pK-CaMV35S-GUS (Figure 5). The pSiM24-GUS/GFP plasmids were also bombarded in onion cells, and strong GUS or GFP expression was observed in transformed onion epidermal cells (Figure 3-4).

### Transformation ability of pSiM24 vector in tobacco and *Arabidopsis*

Tobacco leaf discs were co-cultivated with *A. tumefaciens* for three days, and transformed leaf discs were selected in the presence of 250 mg/L kanamycin and 500 mg/L of cefotaxime for four weeks. The increase in fresh weight in transformed leaf discs was evaluated as described previously [51]. The increases in fresh weight of four-week-old leaf discs were compared and are presented in Table 4. Leaf discs treated with binary vectors showed a seven- to eight-fold increase in fresh weight over the vector-less control, remaining green with multiple regenerating shoots. In the negative vector-less control, leaf discs did not induce callus and turned yellow within two weeks of antibiotic selection. Thus, the percentage of leaf discs showing an increase in fresh weight over the negative vector-less control indicates the proportion of putatively transformed leaf discs, which ranged from 79 to 98%. There appeared to be no detectable difference between pSiM24 binary vectors and the positive control pCAMBIA and pKM24KH vectors. The effect of pSiM24 binary vector on transformation frequency was also studied in *A. thaliana*. The pSiM24 and pSiM24-GUS/GFP vectors exhibited approximately two-fold more transformation frequency in *A. thaliana* than pCAMBIA2300 and pKM24KH vectors (Table 5).

**Table 4:** Binary Ti vectors pSiM24 and pSiM24-GUS/GFP conferred a kanamycin-resistant fresh weight (FW) increase in tobacco leaf discs after transformation with *A. tumefaciens*.

Binary Ti Vectors	Mean FW/plate, g	Increase in FW in g per plate over vector-less control	% Leaf discs with increased FW
Vector-less control	0.83±0.09^a^	0^c^	0
pSiM24	8.12±2.1^b^	7.29±2.13^d^	88
pSiM24-GUS	6.85±1.8^b^	6.02±1.79^d^	98
pSiM24-GFP	7.32±2.4^b^	6.49±2.42^d^	79
pCAMBIA2300	7.85±1.3^b^	7.02±1.33^d^	83
pKM24KH	7.43±1.7^b^	6.6±1.68^d^	81

After the co-cultivation with *A. tumefaciens* strain GV3850 at 25°C for 3 days, leaf discs were selected on shooting medium containing 250 mg/L of kanamycin and 500 mg/L of cefotaxime for four weeks. Each treatment involved 5 plates with 10 leaf discs per plate. The same experiment was repeated two more times. Statistical analysis of the data was performed adopting one way ANOVA analysis (using GraphPad Prism version 5.01) and presented as the means ± S.D. A *P* value of less than 0.05 was considered significant indicated by different superscript letters.

**Table 5:** Effect of binary Ti vectors pSiM24 and pSiM24-GUS/GFP on transformation frequencies in *A. thaliana*.

Binary Ti Vectors	Transformation frequency (%)
Vector-less control	0^a^
pSiM24	2.68±0.29^b^
pSiM24-GUS	2.45±0.26^b^
pSiM24-GFP	2.38±0.21^b^
pCAMBIA2300	1.32±0.12^c^
pKM24KH	1.18±0.2^c^

*A. tumefaciens* harboring binary vectors were cultured and floral dipping of *A. thaliana* plants was subsequently performed as described in materials and methods. T_1_ seedlings were selected on solid MS medium containing kanamycin. The data was obtained from at least four independent lines developed for each construct. Statistical analysis of the data was performed adopting one way ANOVA analysis (using GraphPad Prism version 5.01) and presented as the means ± S.D. A *P* value of less than 0.05 was considered significant indicated by different superscript letters.

### Expression analysis of pSiM24-GUS/GFP in transgenic plants

**Figure 6:**
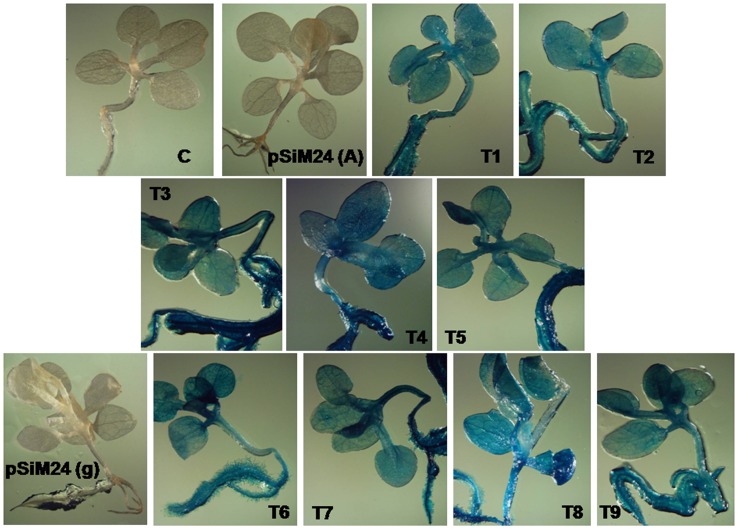
GUS expression in transgenic tobacco plants generated for constructs pSiM24 and pSiM24-GUS. Representative transgenic tobacco plants (second generation, three weeks old) generated by agrobacterium-mediated transformation (T1 to T5 lines) and transformation using gene-gun (T6 to T9 lines) were stained to determine GUS histochemical activity. C: Untransformed tobacco plant; pSiM24 (A): pSiM24 transgenic tobacco plants generated by agrobacterium-mediated transformation; pSiM24 (g): pSiM24 transgenic tobacco plants generated by biolistic bombardment method.

**Figure 7:**
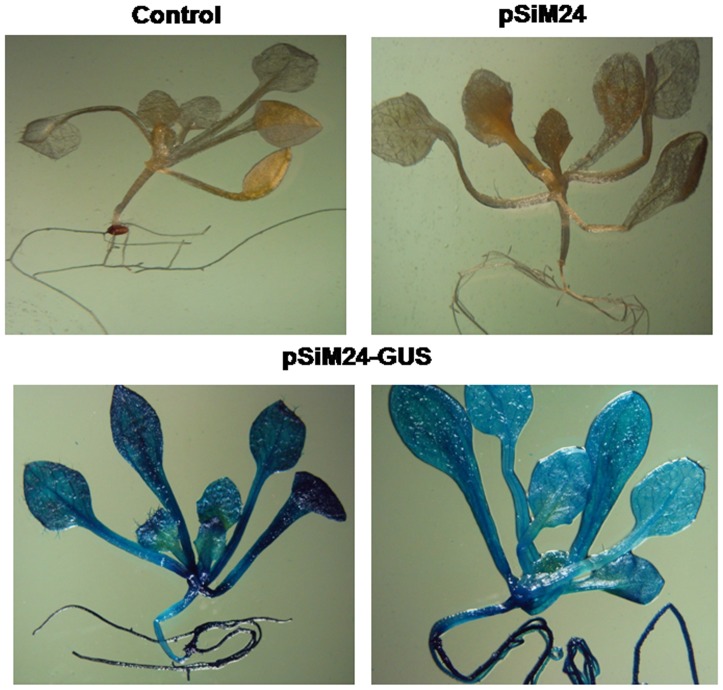
GUS expression in transgenic *Arabidopsis* plants generated for constructs pSiM24 and pSiM24-GUS. Representative transgenic *Arabidopsis* plants (second generation, two weeks old) generated by agrobacterium-mediated transformation were stained to determine GUS histochemical activity. Control: Untransformed *Arabidopsis* plant; pSiM24: pSiM24 transgenic *Arabidopsis* plants; pSiM24-GUS: pSiM24-GUS transgenic *Arabidopsis* plants.

**Figure 8:**
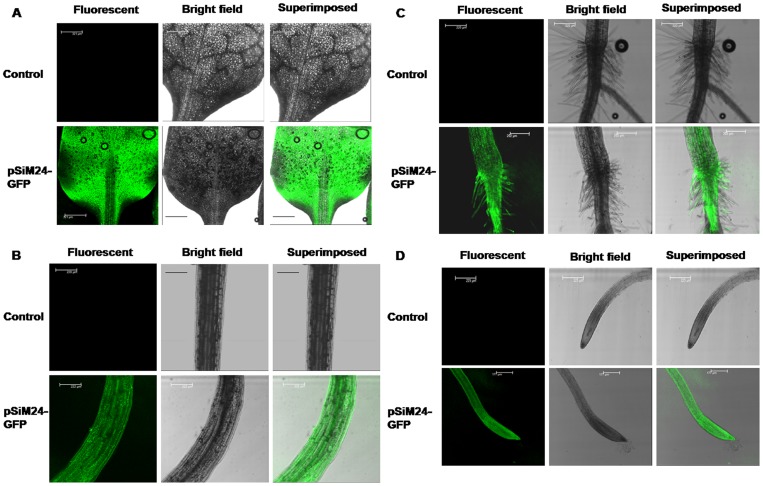
GFP expression in leaf, stem and roots of transgenic *Arabidopsis* plants generated for construct pSiM24-GFP. (A) Representative transgenic *Arabidopsis* plant leaves (second generation, two weeks old) generated by agrobacterium-mediated transformation were imaged to determine GFP activity. Fluorescent, bright-field and superimposed (bright-field and green fluorescent) confocal laser scanning micrographs of transgenic *Arabidopsis* leaves. Scale bar represents 320 µm. (B) Representative transgenic *Arabidopsis* plant stems (second generation, two weeks old) generated by agrobacterium-mediated transformation were imaged to determine GFP activity. Fluorescent, bright-field and superimposed (bright-field and green fluorescent) confocal laser scanning micrographs of transgenic *Arabidopsis* stems. Scale bar represents 220 µm. (C) Representative transgenic *Arabidopsis* plant stem-root junctions (second generation, two weeks old) generated by agrobacterium-mediated transformation were imaged to determine GFP activity. Fluorescent, bright-field and superimposed (bright-field and green fluorescent) confocal laser scanning micrographs of transgenic *Arabidopsis* stem-root junctions. Scale bar represents 220 µm. (D) Representative transgenic *Arabidopsis* plant roots (second generation, two weeks old) generated by agrobacterium-mediated transformation were imaged to determine GFP activity. Fluorescent, bright-field and superimposed (bright-field and green fluorescent) confocal laser scanning micrographs of transgenic *Arabidopsis* roots. Scale bar represents 225 µm.

**Figure 9:**
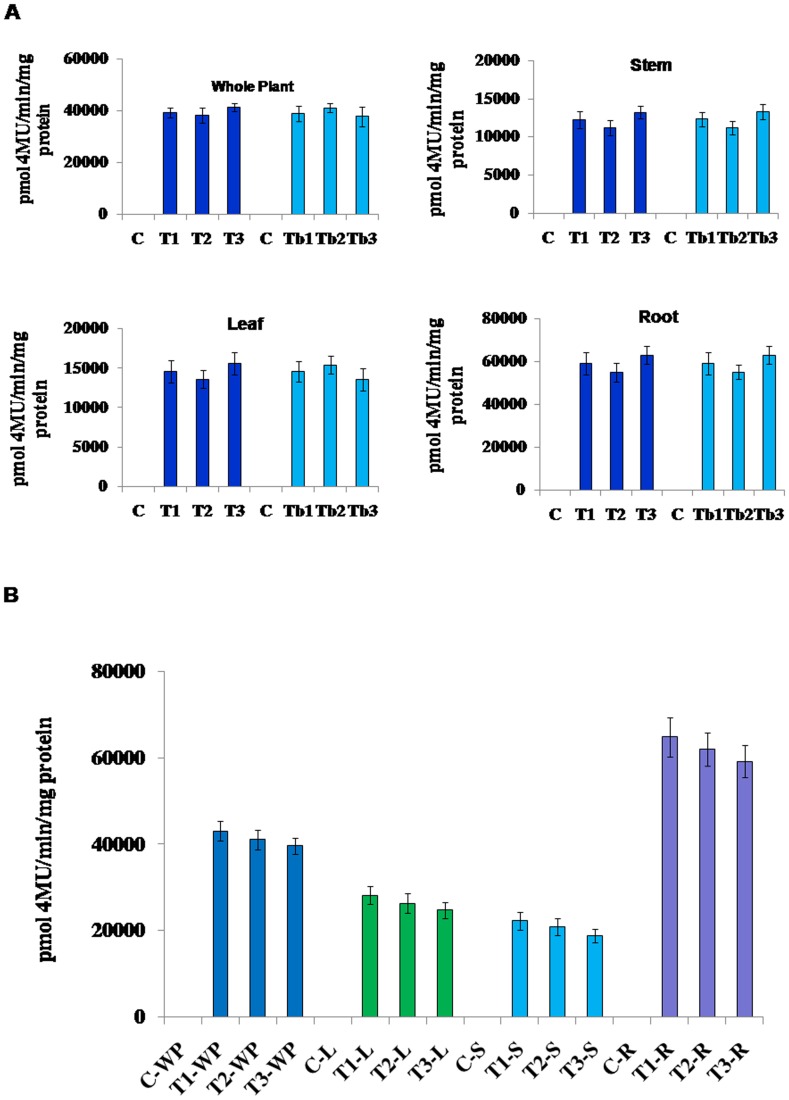
GUS expression in transgenic tobacco and *Arabidopsis* plants generated for constructs pSiM24 and pSiM24-GUS. (A) GUS enzymatic activity of pSiM24-GUS transgenic tobacco (second generation, 3 weeks old) lines was measured in whole plant, leaf, stem and root tissues. Soluble protein extracts isolated from different tissues of plants were used for GUS assay along with the wild-type plants (C). The data represent means ± S.D. of four second generation individuals from one line for each tissue (n = 4). The values significantly differ between control and transgenic plants at P<0.01 based on Student's *t*-test. T1, T2 and T3: Representative transgenic lines generated by agrobacterium-mediated plant transformation procedure; Tb1, Tb2 and Tb3: Representative transgenic lines generated by biolistic plant transformation procedure. (B) GUS enzymatic activity of pSiM24-GUS transgenic *Arabidopsis* (second generation, two weeks old) lines was measured in whole plant (WP), leaf (L), stem (S) and root (R) tissues. Soluble protein extracts isolated from different tissues of plants were used for GUS assay along with the wild-type plants (C). The data represent means ± S.D. of four second generation individuals from one line for each tissue (n = 4). The values significantly differ between control and transgenic plants at P<0.01 based on Student's *t*-test. T1, T2 and T3: Representative transgenic lines generated by floral-dip plant transformation procedure.

**Figure 10:**
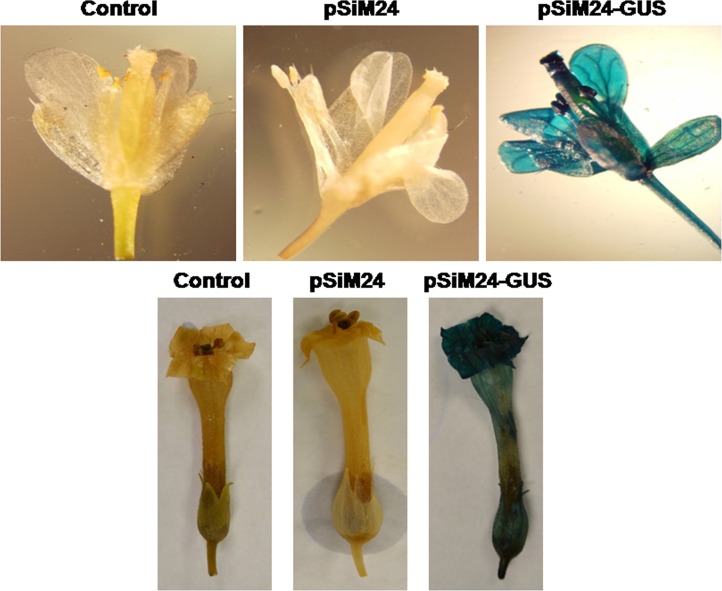
GUS expression in flowers of transgenic tobacco and *Arabidopsis* plants generated for constructs pSiM24 and pSiM24-GUS. Histological GUS staining in different floral tissues of untransformed (control), pSiM24 and pSiM24-GUS plants. Histological GUS staining shows strong GUS expression in all floral tissues of transgenic pSiM24-GUS *Arabidopsis* (upper panel) and tobacco (lower panel) plants.

*Agrobacterium* carrying the pSiM24-GUS reporter gene was used to transform *Arabidopsis* and tobacco plants. GUS histochemical analysis confirmed that the pSiM24 vector successfully expressed GUS genes in transgenic *Arabidopsis* and tobacco (both by agrobacterium-mediated transformation as well as by gene-gun methods) plants (Figure 6–7). The *Arabidopsis* pSiM24-GUS and pSiM24-GFP transgenic plants successfully expressed GUS and GFP proteins, as detected by GUS histochemical staining and confocal microscopy of GFP (Figure 7–8). Furthermore, GUS analysis of second-generation plants confirmed the successful inheritance of the transgene from one generation to another generation in both transgenic tobacco and *Arabidopsis* plants (Figure 9–10). The GUS activity was estimated biochemically in R2-generation transgenic *Arabidopsis* and tobacco plants; it was observed that approximately two times higher GUS activity accumulated in leaf and stem tissues of *Arabidopsis* plants than in tobacco plants (Figure 9). The expression of GUS activities in different tissues of transgenic *Arabidopsis* and tobacco plants containing pSiM24-GUS showed the following pattern: Root > Leaf > Stem (Figure 9). In addition, the histological GUS staining documented that the level of GUS expression was high in the reproductive tissues of transgenic pSiM24-GUS tobacco and *Arabidopsis* plants (Figure 10). Both in tobacco and *Arabidopsis* transgenic plants, GUS transcript levels were higher for the pSiM24 binary vector than for the pKYLX-based expression vector pKCaMV35 (Figure 5).

### Transient expression of GUS using pSiM24 vector through vacuum infiltration method

**Figure 11:**
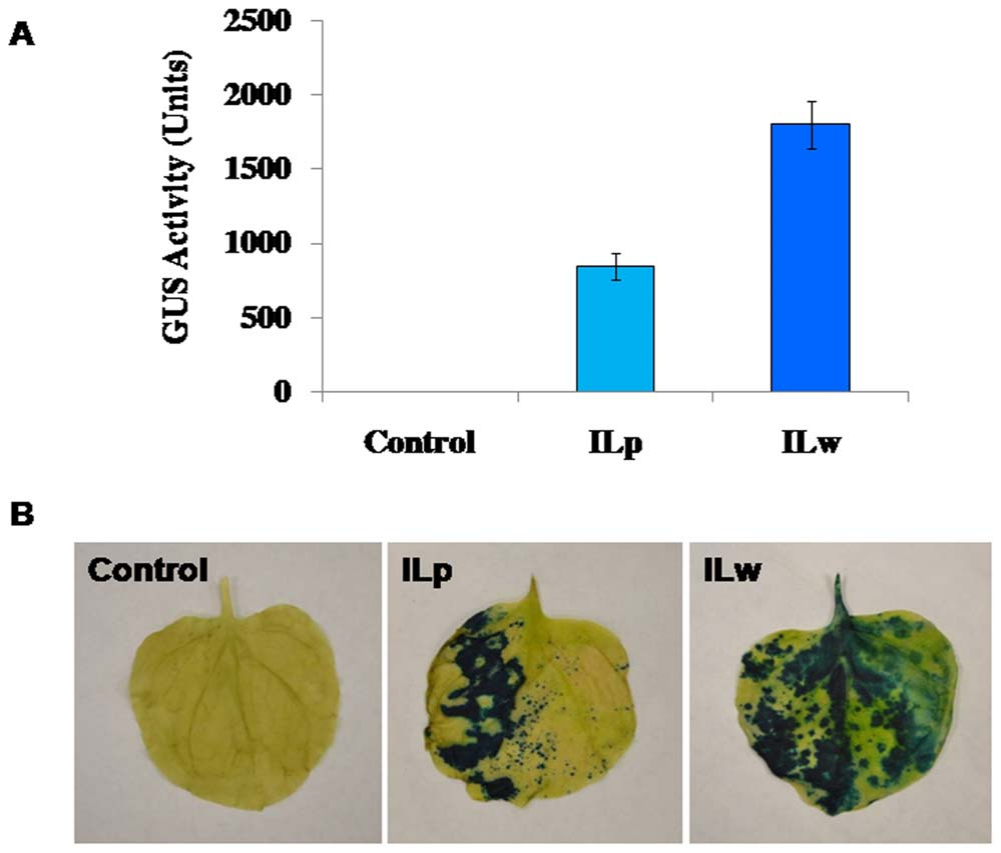
Transient expression of GUS in pSiM24-GUS Agro-infiltrated *N. benthamiana* leaves using vacuum infiltration method. (A) GUS enzymatic activity of pSiM24 and pSiM24-GUS *A. tumefaciens*-infiltrated *N. benthamiana* leaves was measured, and one unit of GUS activity was defined as the amount of enzyme that liberated 1 pmol 4-methylumbelliferone mg^−1^ protein min^−1^. The data represent means ± S.D. of four biological replicates for each construct (n = 4). The values significantly differ between control (pSiM24) and pSiM24-GUS agro-infiltrated leaf samples at P<0.01 based on Student's *t*-test. ILw: Whole Infiltrated Leaf; ILp: Partial Infiltrated Leaf. (B) The pSiM24 and pSiM24-GUS *A. tumefaciens*-infiltrated *N. benthamiana* leaves were histochemically stained for GUS enzyme. The pSiM24-GUS agro-infiltrated leaves showed stronger GUS expression, as detected by histochemical staining. Control: *N. benthamiana* leaf infiltrated with *A. tumefaciens* harboring pSiM24 construct; ILw: Whole Infiltrated Leaf; ILp: Partial Infiltrated Leaf. Both ILw and ILp are *N. benthamiana* leaves infiltrated with *A. tumefaciens* carrying pSiM24-GUS construct.

**Figure 12:**
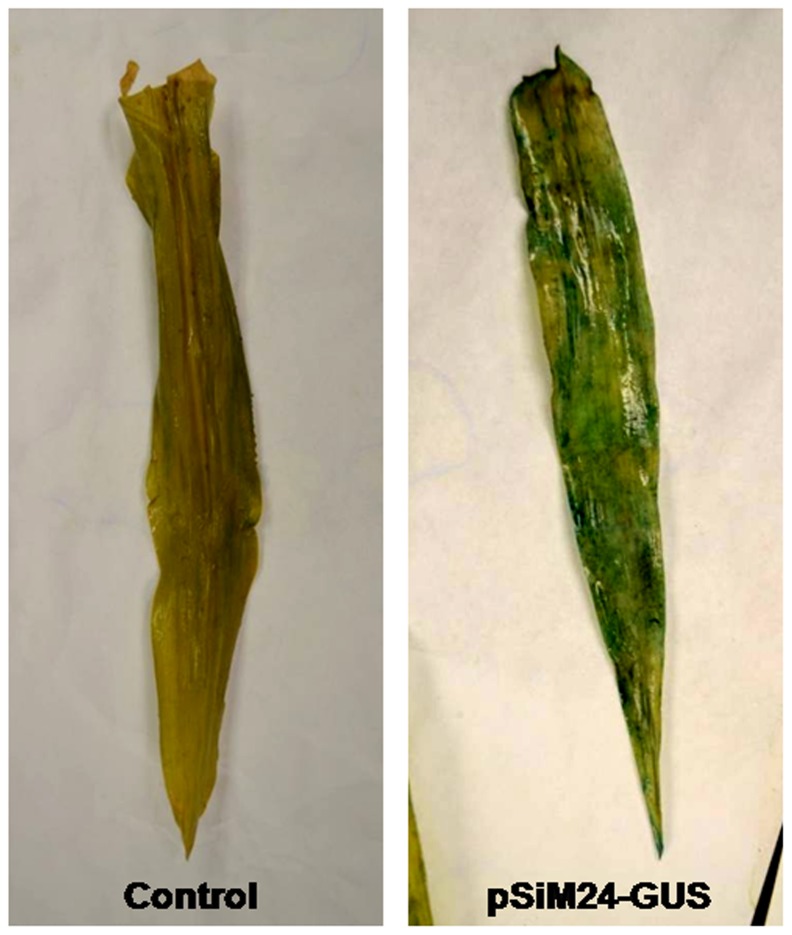
Transient expression of GUS in pSiM24-GUS Agro-infiltrated *Zea mays* leaves using vacuum infiltration method. The representative pSiM24-GUS agro-infiltrated leaf showed strong GUS expression, as detected by histochemical staining. Control: *Z. mays* leaf infiltrated with *A. tumefaciens* carrying pSiM24; pSiM24-GUS: *Z. mays* leaf infiltrated with *A. tumefaciens* harboring pSiM24-GUS construct.

*A. tumefaciens* carrying pSiM24 and pSiM24-GUS constructs infiltrated *N. benthamiana* leaves were assayed and histochemically stained for GUS enzyme. The completely infiltrated leaves showed approximately 1800 GUS units, whereas the partially infiltrated leaves exhibited approximately 850 GUS units (Figure 11A). One unit of GUS activity was defined as the amount of enzyme that liberated 1 p mol 4-methylumbelliferone mg^−1^ protein min^−1^ [52]. The agro-infiltrated leaves showed strong GUS expression, as detected by histochemical staining, in leaves of both *N. benthamiana* and *Zea mays* (Figure 11B and Figure 12).

### Transient expression of GFP*-AtCESA3^ixr1-2^*, Vip3A(a), KMP-11, IL-10 and nat-T-phyllo-GFP genes using pSiM24 vector

**Figure 13:**
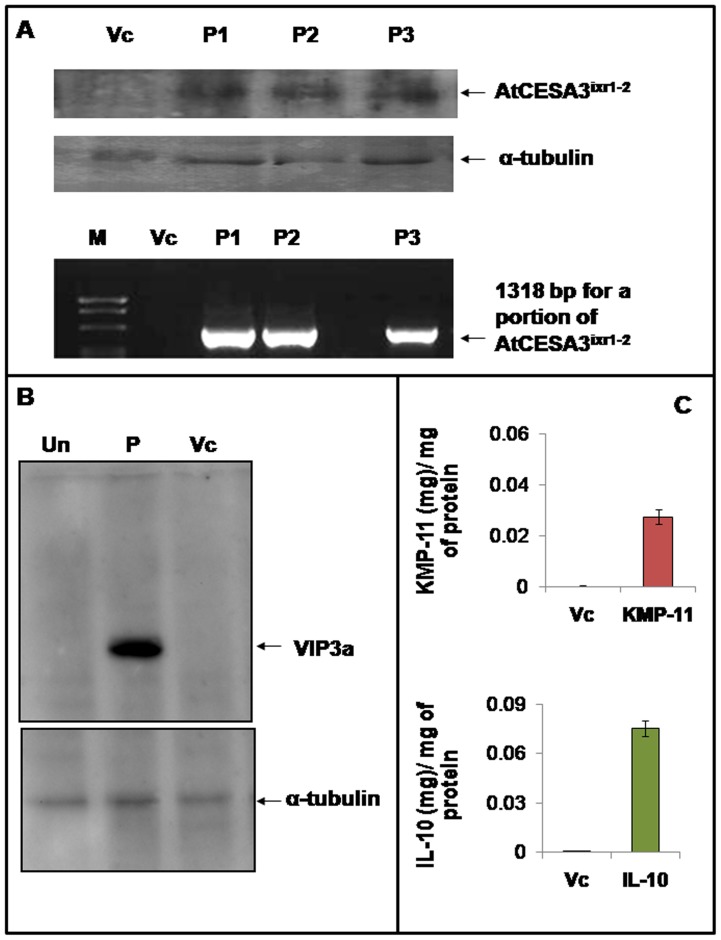
Transient expression of *GFP-AtCESA3^ixr1-2^*, Vip3A(a), KMP-11 and IL-10 genes using pSiM24 expression vector. (A) Western blot analysis of transiently expressed *GFP* fused *Arabidopsis CESA3^ixr1-2^* (*GFP-AtCESA3^ixr1-2^*) detected with AtCESA3-specific polyclonal antibodies showed the expected bands of size 145 kD (Upper panel). Signals were quantitated, normalized to the α-tubulin loading control (Middle panel). RT-PCR products for a portion of *GFP-AtCESA3^ixr1-2^* with the expected 1318 bp band are shown (Lower panel). Transiently *GFP-AtCESA3^ixr1-2^* expressed agro-infiltrated *N. tabacum* L. variety Samsun NN leaf samples using pSiM24-*GFP-AtCESA3^ixr1-2^* (P1, P2 and P3) and pSiM24 (Vc; empty vector control) are presented. (B) Western blot of vegetative insecticidal protein, Vip3A(a) expression in tobacco protoplast. Detection by Vip3A-specific polyclonal antibodies showed the expected bands of size 88 kD (Upper panel). Un: Untransfected control; P: pSiM24-Vip3A(a) transfected protoplast; Vc: pSiM24 vector control transfected protoplast. Signals were quantitated, normalized to the α-tubulin loading control (Lower panel). (C) Estimation of KMP-11 (Kinetoplastid membrane protein-11) concentration (expressed as mg KMP-11 per mg protein) in tobacco protoplasts by ELISA. KMP-11: pSiM24-KMP-11 transfected protoplast; Vc: pSiM24 vector control transfected protoplast. (D) Estimation of Interleukin-10 (IL-10) concentration (expressed as mg IL-10 per mg protein) in tobacco protoplasts by ELISA. IL-10: pSiM24-IL-10 transfected protoplast; Vc: pSiM24 vector control transfected protoplast.

**Figure 14:**
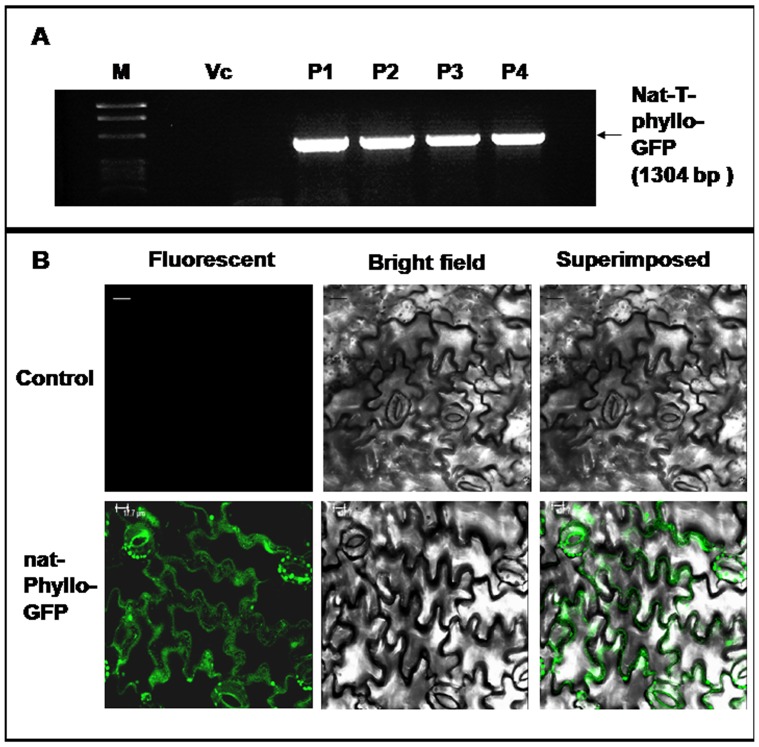
Transient expression of *nat-T-phyllo-GFP* using pSiM24 expression vector. (A) RT-PCR products for native T-phylloplanin fused GFP (*nat-T-phyllo-GFP*) with the expected 1304 bp band are shown. Transiently *nat-T-phyllo-GFP* expressed agro-infiltrated *N. tabacum* L. variety Samsun NN leaf samples using pSiM24-*nat-T-phyllo-GFP* (P1, P2, P3 and P4) and pSiM24 (Vc; empty vector control) are presented. (B) Localization analysis of apoplast targeted nat-T-phyllo-GFP by confocal laser scanning microscopy. Agro-infiltrated *N. tabacum* L. variety Samsun NN leaf cells for pSiM24-nat-T-phyllo-GFP construct expressing GFP (green fluorescence) was visualized by confocal laser scanning microscopy. No GFP fluorescence was detected in agro-infiltrated tobacco leaf cells for pSiM24 construct (Control). Scale bar represents 20 µm on all images. Fluorescent, bright-field and superimposed (bright-field and green fluorescent) confocal laser scanning micrographs of leaf sections are presented.

Western blot analysis of pSiM24-GFP*-AtCESA3^ixr1-2^* agroinfiltrated leaf samples showed the expected bands of size 145 kD for *GFP-AtCESA3^ixr1-2^* as detected with AtCESA3-specific polyclonal antibodies (Figure 13A). In addition, RT-PCR analysis of agroinfiltrated leaf samples exhibited expected 1318 bp band for a portion of *GFP-AtCESA3^ixr1-2^* (Figure 13A). The transient expression of Vip3A(a) using pSiM24 expression vector in tobacco protoplast was detected by Vip3A-specific polyclonal antibodies that showed the expected bands of size 88 kD (Figure 13B). Using pSiM24 expression vector KMP-11 and IL-10 were also expressed transiently in tobacco protoplasts and showed expression up to 0.03 mg of KMP-11 and 0.08 mg of IL-10 per mg of protoplast protein samples by indirect ELISA (Figure 13C). The RT-PCR analysis and localization analysis of apoplast targeted nat-T-phyllo-GFP by confocal laser scanning microscopy showed the successful expression of *nat-T-phyllo-GFP* using pSiM24 expression vector (Figure 14).

## Discussion

A binary vector, used as a standard tool in the transformation of higher plants mediated by *A. tumefaciens*, consists of T-DNA and the vector backbone. T-DNA is the segment delimited by the border sequences, the right border (RB) and the left border (LB), and may contain multiple cloning sites, a selectable marker gene for plants, a reporter gene and other genes of interest [7], [53]. The vector backbone carries plasmid replication functions for *E. coli* and *A. tumefaciens*, selectable marker genes for the bacteria, optionally a function for plasmid mobilization between bacteria and other accessory components [7], [8].

The binary vector pSiM24 has an overall size of 7.08 kb and carries a plant-gene expression cassette containing a highly active, constitutive promoter (M24) (GenBank Accession no. KF032933). The size of the pSiM24 vector is approximately 2000 bp shorter than the commercially available pCAMBIA vectors (www.cambia.org) and approximately 6000 bp shorter than pKYLX-based vectors [4]. In the pSiM24 binary vector, only the necessary elements were included to attain a minimum size. The right border (RB) and the left border (LB) of pSiM24 are imperfect, direct repeats of 25 bases. The RB and LB are considered to be the only essential cis-elements for T-DNA transfer [54]. The promoter carried by the expression cassettes described here has been studied in transgenic plants (present study) and is also functional in plants such as tobacco [41]–[44], [55], *Arabidopsis* and corn (Sahoo and Maiti, Unpublished Data). It has been documented that the *Mirabilis mosaic virus* full-length transcript promoter is constitutive in nature, exhibiting 14 to 25 times stronger activity than CaMV35S in the tobacco protoplast transient system and transgenic tobacco plants, respectively [27], [34], [42]. The modified full-length transcript promoter (M24) of the *Mirabilis mosaic virus* with duplicated enhancer domains [27], [29], [41]–[44], [55] was used in the pSiM24 vector to evaluate gene expression in plants. In the pSiM24-GUS vector, the coding sequence of GUS was placed between the heterologous M24 promoter and the terminator sequence from the rbcSE9 gene (Figure 1) [43]–[44]. We showed that microT-DNAs in pSiM24 containing a kanamycin resistance gene and reporter gene (GUS or GFP) were integrated stably in the nuclear chromosomal DNA of transgenic plants for successive generation.

Selectable markers need to be expressed in calli, in cells from those plants that are being regenerated or in germinating embryos to facilitate plant transformation. Therefore, promoters for constitutive expression are preferred. In pSiM24, the Nos promoter derived from nopaline synthase (Nos) of *A. tumefaciens* [56] was used to express the selectable marker gene (Figure 1). The choice of promoters responsible for selectable marker gene expression also plays an important role in the efficiency of transformation [57]–[59]. The use of weak promoters may not always be a bad idea because the levels of expression of marker genes and genes of interest are often linked, and the selection of transformants with weak selectable markers may cause strong expression of the gene of interest to be obtained [8]. It is generally recommended that different promoters be used for the selectable marker and expressing the gene of interest [57]–[59], as in the pSiM24 vector (Figure 1), which carries the M24 promoter for the expression of the gene of interest (here GUS in pSiM24-GUS) and Nos for the selectable marker (here, KanR). Homology-based gene silencing has been reported to occur extensively in transgenic plants [60]. Gene silencing due to promoter homology can be avoided by either using diverse promoters isolated from different plant and viral genomes or by designing synthetic promoters [27], [28], [34], [38], [61]–[68].

Depending on the plant species to be transformed, the choice of selectable markers greatly affects the efficiency of transformation, and permissive concentrations of selective agents vary considerably among plant species. Genes resistant to antibiotics or herbicides, such as kanamycin, hygromycin, phosphinothricin and glyphosate, are very popular. Kanamycin resistance has been most frequently exploited in the transformation of many dicotyledonous plants such as tobacco, tomato, potato and *Arabidopsis* [34], [42], [69]. The pSiM24 binary vector contains a synthetic ‘nptII’ KanR gene (nos promoter-KanR cDNA-Nos terminator), the open reading frame of which is optimized for plant codon bias; hence, the nptII gene serves both as a selectable marker for the regeneration of plantlets on kanamycin-containing medium (for tobacco 250–300 µg/ml) and as a screenable marker for agrobacterium (25 µg/ml). In the present study, pSiM24-containing nptII gene was used to select the transformed *Arabidopsis* and tobacco plants in 30 µg/ml and 250 µg/ml kanamycin, respectively, (Figure 6–7). Choice of antibiotics is an important factor in plant transformation. For example, kanamycin may not suitable for rice and maize cells, whereas hygromycin resistance (hpt) is very good for rice transformation [10], and the phosphinothricin resistance gene (bar) is efficient for maize and other cereals [70], [71]. We also developed a binary vector pKDH, which has a structure similar to that of pSiM24, but the selection marker KanR gene was replaced with a hygromycin resistance (HygR, Hygromycin B transferase, HPH) gene for the selection of transgenic monocot plants, and the sequence information of the binary vector pKDH was provided (Genebank Accession no. KF041008).

The components of the pSiM24 expression system vector are arranged in a modular configuration in which the promoter, terminator and MCS cassettes are flanked by unique restriction endonuclease cleavage sites. The pSiM24 vector provides nine unique cloning sites in the first multiple cloning site (MCS) between the left T-DNA border and the M24 promoter (BstXI, StuI, EspAI, PasI, KflI, Bstz17I, SmaI, XmaI and EcoRI), twelve unique cloning sites in the second MCS between the M24 promoter and the pea rbcSE9 terminator (HindIII, AbsI, PspXI, SciI, XhoI, HpaI, MluI, Eco53kI, SacI, SbfI, PstI, and XbaI) and seven unique cloning sites in the third MCS between the Nos promoter and the right T-DNA border (BglII, BstEII, EcoNI, FseI, SwaI, NruI, and PacI). This configuration facilitates the modification or replacement of individual components in the pSiM24 vector. The MCS in pSiM24 contains more additional cleavage sites than that of pUC19. It should be noted that the orientation of the MCS in the pSiM24 plasmid, relative to the rbcS and M24 promoters, is opposite that in pUC19, relative to the lac promoter. The presence of a number of cloning sites unique to the three MCS allow for gene-stacking applications to introduce multiple gene with additional sequences, such as translational initiation, signal and transit peptide sequences and translational termination, into these plasmids. The pSiM24 vector provides a number of options for cloning, transformation and expression strategies. The M24 promoter in the pSiM24 plasmid can be easily replaced with other promoters as EcoRI-HindIII cassettes, thus making different strategies for the regulated expression of foreign genes possible.

Reporter genes, whose expression can be easily monitored, are useful in many ways in plant transformation. Strength and temporal, spatial and other types of regulation of promoters and elements may be conveniently assayed by connecting these elements to the reporter genes. Genes for β-glucuronidase (GUS) [9], luciferase [72] and GFP [73] are popular examples. In the present study, two different reporter genes, GUS and GFP, were introduced into the pSiM24 vector to monitor and analyze their expression under the M24 promoter in both stable and transient systems. Reporter genes that are connected to constitutive promoters may be used to monitor the process of transformation. The expression of the reporter genes soon after the inoculation of plant cells with *A. tumefaciens*, which is referred to as “transient expression”, is a good indication of the transfer of the T-DNA from the bacteria to the nuclei of plant cells. The expression of the reporter genes later in a cluster of cells growing on selection media provides evidence of the integration of the T-DNA in plant chromosomes. A binary vector that carries a constitutive selectable marker and a constitutive reporter is very useful as a control vector both in transformation experiments and in assays of gene expression. Hence, in pSiM24, both “nptII” and GUS/GFP were constitutively expressed by using two different constitutive promoters, i.e., Nos for nptII and M24 for GUS/GFP, for expression in transgenic plants (Figure 6–10).

The rbcSE9 polyadenylation signal used in the pSiM24 vector has previously been used to direct efficient mRNA3′ end formation from chimeric genes in transformed tobacco [74]–[76]. These 3′ ends are identical to those observed in pea, indicating that this signal is suitable for the predictable expression of foreign genes in plants. The 3′ regions of the cauliflower mosaic virus 35S transcript and the nopaline synthase gene in the wild-type T-DNA of *A. tumefaciens* are frequently used as a 3′ signal to direct selectable marker genes expression.

In pSiM24, the “bla” AmpR gene, which confers resistance to ampicillin, was used as the marker for bacterial selection for *E. coli*. The selectable marker for plants, Nos-nptII, in pSiM24 also provides fair levels of resistance to both *E. coli* and *A. tumefaciens*. Binary vectors need to be replicated both in *E. coli* and *A. tumefaciens*. Hence, the pSiM24 vector carries all of the functions necessary for replication and transfer in *Escherichia coli* and *A. tumefaciens*, which includes a ColE1-replicon and an RK2-replicon derived from pRK2013 [77]. The pSiM24 binary vector carries the origin of vegetative replication (OriV) and the transacting replication functions (Trf) of plasmid incompatibility group P (IncP) plasmids [78]. The types of replication functions exploited determine the copy numbers and stability of the plasmids in bacterial cells. *E. coli* exhibited a transformation frequency up to five- to six-fold higher with pSiM24 than with conventional pCAMBIA and pKM24KH vectors, (Table 1) and the plasmid DNA yields of pSiM24 binary Ti vectors were three-fold and seven- to eight-fold higher in *E. coli* than those of conventional pCAMBIA 2300 and pKM24KH, respectively (Table 3). The pSiM24 binary vector contains the ColE1 replicon without a bom (basis of mobility) sequence, which again reduces its size. The bom function is necessary for plasmid mobilization from *E. coli* to *A. tumefaciens* [79]. This function is not necessary when vectors are introduced into *A. tumefaciens* by electroporation or freeze-thaw methods.

Not only reporter genes, other introduced genes of size up to 4 kb were also effectively expressed using pSiM24 expression vector. In the present study, nat-T-phyllo-GFP [44] was expressed transiently using pSiM24 expression vector in tobacco leaves. T-phylloplanins have antimicrobial properties and are known to inhibit blue mold disease caused by *Peronospora tabacina* [41], [44], [80], [81]. Both the native and mature tobacco phylloplanin gene fused with GFP targeted to the apoplasm increases resistance to blue mold disease in tobacco [41], [44]. Here, the expression of nat-T-phyllo-GFP using pSiM24 vector was confirmed by the GFP fluorescence in apoplast region of agroinfiltrated plant leaves (Figure 14). Another, chimeric gene (GFP*-AtCESA3^ixr1-2^*) of size 4086-bp fragment was also successfully expressed transiently using pSiM24 following agro-infiltration procedure. The overexpression of GFP fused to the *Arabidopsis CESA3^ixr1-2^* (GFP*-AtCESA3^ixr1-2^*) gene in transgenic tobacco was known for increasing cellulose digestibility and biomass saccharification [42], [43]. Further genes like Vip3A(a), KMP-11 and IL-10 were also successfully expressed transiently in tobacco protoplasts using pSiM24 expression vector (Figures 13). The gene product of novel vegetative insecticidal gene, vip3A(a) shows activity against lepidopteran insects [45], [46]. KMP-11, a flagellar protein is known to play an essential role in regulating cytokinesis in both amastigote and promastigote forms of leishmania [47] and is a potential stimulator of T-lymphocyte proliferation [82]. Interleukin-10 (IL-10), an anti-inflammatory cytokine secreted under different conditions of immune activation by a variety of cell types, including T cells, B cells, and monocytes/macrophages [83], [84] has been shown to suppress a broad range of inflammatory responses and as an important factor in maintaining homeostasis of overall immune responses [85], [86] and thus has been used for developing novel therapies for several human diseases such as allergic responses and autoimmune diseases [87].

The effect of pSiM24 binary vector on transformation frequency studied in *A. thaliana* verified the pSiM24 and pSiM24-GUS/GFP vectors exhibited more transformation frequency in *A. thaliana* than pCAMBIA2300 and pKM24KH vectors (Table 5) however strong GUS transgene expression in pSiM24-GUS transgenic tobacco and *Arabidopsis* than pK-CaMV35S-GUS transgenic plants depends upon the strong M24 promoter (Figure 5) [41], [42].

The pSiM24 vector was observed to be active in transferring the transgene both transiently (Figure 3–5, Figure 11–14) and stably (Figure 6–10) in plant systems, making it useful for various plant biotechnological applications. This plasmid has multiple cloning sites and can act as a platform for various applications, such as gene expression studies and different promoter expressional analyses. In addition, pSiM24 offers a wide selection of cloning sites and high copy numbers in *E. coli* for the facile manipulation of different genetic elements. Thus, the pSiM24 binary vector system described in this study has a high degree of flexibility and may serve as a useful tool for the transformation of plants, making it apt for future use in field release experiments.
